# Immunomodulating Activity of *Pleurotus eryngii* Mushrooms Following Their In Vitro Fermentation by Human Fecal Microbiota

**DOI:** 10.3390/jof8040329

**Published:** 2022-03-22

**Authors:** Marigoula Vlassopoulou, Nikolaos Paschalidis, Alexandros L. Savvides, Georgia Saxami, Evdokia K. Mitsou, Evangelia N. Kerezoudi, Georgios Koutrotsios, Georgios I. Zervakis, Panagiotis Georgiadis, Adamantini Kyriacou, Vasiliki Pletsa

**Affiliations:** 1 Institute of Chemical Biology, National Hellenic Research Foundation, 11635 Athens, Greece; mvlassopoulou@eie.gr (M.V.); panosg@eie.gr (P.G.); 2 Department of Nutrition and Dietetics, Harokopio University, 17671 Athens, Greece; gsaxami@hua.gr (G.S.); emitsou@hua.gr (E.K.M.); dp4421804@hua.gr (E.N.K.); mkyriacou@hua.gr (A.K.); 3 CyTOF Laboratory, Biomedical Research Foundation of the Academy of Athens (BRFAA), 11527 Athens, Greece; nikpaschal@gmail.com; 4 Microbiology Group, Department of Botany, Faculty of Biology, National and Kapodistrian University of Athens, Panepistimioupolis, Zografou, 15781 Athens, Greece; alsavvi@biol.uoa.gr; 5 School of Medical Sciences, Örebro University, SE-701 82 Örebro, Sweden; 6 Laboratory of General and Agricultural Microbiology, Department of Crop Science, Agricultural University of Athens, 11855 Athens, Greece; georgioskoutrotsios@gmail.com (G.K.); zervakis@aua.gr (G.I.Z.)

**Keywords:** edible mushrooms, macrophages, cytokines, PBMCs

## Abstract

Recent studies have revealed the crucial role of several edible mushrooms and fungal compounds, mainly polysaccharides, in human health and disease. The investigation of the immunomodulating effects of mushroom polysaccharides, especially β-glucans, and the link between their anticancer and immunomodulatory properties with their possible prebiotic activity on gut micro-organisms has been the subject of intense research over the last decade. We investigated the immunomodulating effects of *Pleurotus eryngii* mushrooms, selected due to their high β-glucan content, strong lactogenic effect, and potent geno-protective properties, following in vitro fermentation by fecal inocula from healthy elderly volunteers (>60 years old). The immunomodulating properties of the fermentation supernatants (FSs) were initially investigated in U937-derived human macrophages. Gene expression as well as pro- (TNF-α, IL-1β) and anti-inflammatory cytokines (IL-10, IL-1Rα) were assessed and correlated with the fermentation process. The presence of *P. eryngii* in the fermentation process led to modifications in immune response, as indicated by the altered gene expression and levels of the cytokines examined, a finding consistent for all volunteers. The FSs immunomodulating effect on the volunteers’ peripheral blood mononuclear cells (PBMCs) was verified through the use of cytometry by time of flight (CyTOF) analysis.

## 1. Introduction

Edible mushrooms have been used for centuries in traditional medicine as enhancers of longevity and well-being [1]. Current research has identified many of their health-promoting properties, ranging from antioxidant, antimicrobial, and anticancer activities to immune enhancement and prebiotic action [2,3]. These beneficial effects have been attributed to a plethora of biomolecules that are found in mushrooms, especially polysaccharides, with β-glucans being in the spotlight lately, due to their possible prebiotic activity on gut microorganisms [4].

Emphasis has also been placed on the immunomodulating effects of mushroom polysaccharides, especially β-glucans [5], which were investigated, mainly in vitro, using immunocompetent cells, such as monocytes, dendritic cells, and macrophages [6,7,8]. In addition, fungal polysaccharides are suggested to enhance cell-mediated immune responses in vivo and in vitro, and act as biological response modifiers, while even their anticancer properties have been linked to immunomodulatory effects rather than direct cytotoxicity [6,7].

β-(1,3)-d-Glucans and β-(1,3)/(1,6)-d-glucans are essential constituents of the cell wall of basidiomycetes [7], and differ widely in the ratio and arrangement of the 1,3-and 1,6-β-glycoside bonds [9]. Recently, mushrooms’ β-glucans have attracted significant interest towards the development of functional foods [10] as they are known tο exert a direct as well as indirect impact on the immune system. They are directly recognized by various receptors, mostly by Dectin-1 and complement receptor (CR) 3 (CD11b/CD18), present on membranes of cells such as macrophages, monocytes, dendritic cells, and natural killer (NK) cells. Upon binding, direct receptor and/or cellular pathway activation occur, resulting, among others, in cytokine production and antibody responses [11]. When β-glucans end up undigested in the colon, they are ultimately fermented by the intestinal microbiota into short chain fatty acids (SCFAs), mainly acetate, propionate, and butyrate, which have an immunomodulatory activity and are indispensable for the maintenance of the immune homeostasis not only in the intestine but also in several other systems of the human body [12,13]. β-Glucans’ fermentation in the colon often leads to the increase of the populations of beneficial bacteria, such as Bifidobacteria and Lactobacilli; thus, β-glucans are considered potential prebiotics [14] able to affect the composition of gut microbiota (GM), i.e., of a microbial community, playing an important role in maintaining host organism homeostasis through a variety of mechanisms that have not yet been elucidated [15]. The intestinal microbiota interact with the diet, the host’s epithelium, and the immune system; provide protection against invasion of pathogens; participate in the regulation of various metabolic pathways; and ultimately induce significant alterations in the host’s physiology [16].

The link between the genoprotective, anticancer, and immunomodulatory properties of mushrooms, and β-glucans, with their possible prebiotic activity on gut microorganisms, remains a central question and has been the subject of intense research over the last decade [8,17,18,19,20,21,22,23]; nevertheless, the potential genoprotective properties of edible mushrooms within the frame of their fermentation by intestinal microbiota has been addressed by a few studies that have provided encouraging albeit inconclusive results [24,25,26,27,28]. Very recently, the genoprotective action of edible mushrooms of the genus *Pleurotus* (Basidiomycota, Pleurotaceae) was documented for the first time; supernatants deriving from their in vitro fermentation by fecal inocula from elderly (>60 years), healthy volunteers exhibited a clear protective effect against the oxidative agent tert-butyl hydroperoxide (t-BOOH)-induced DNA damage [29]. This finding provided substantial evidence that edible mushrooms may contain ingredients protecting genome integrity, which is of fundamental importance, especially for healthy ageing [29].

In Greece, investigations on the diversity of macrofungi have intensified over the last 20 years, and to date, more than 2800 species have been recorded [30]; among them, selected species are studied both for their use in biotechnological applications and for the production of mushrooms with improved organoleptic, functional, and nutritional properties [10,31,32,33]. Within this frame, we previously investigated the lactogenic, prebiotic [34], and genoprotective effects [29] of *Pleurotus eryngii*, *Pleurotus ostreatus*, and *Cyclocybe cylindracea*, after having screened several strains of basidiomycetes with respect to their β-glucan content and mushroom cultivation performance [29,32], following their in vitro fermentation by fecal inocula from healthy elderly volunteers (>60). In parallel, we assessed the global metabolic profile of the pre- and post-fermentation supernatants (FSs) by the use of proton nuclear magnetic resonance (^1^H-NMR) spectroscopy in order to establish metabolic biomarkers of the fermentation process relevant to human health [29]. Having met all criteria, the fermentation supernatants of *P. eryngii* were selected for further investigation regarding immunomodulatory activity.

*P. eryngii*, also known as the “king oyster mushroom”, is mainly cultivated in Europe, North America, and in many parts of Asia. Fruitbodies are rich in proteins, carbohydrates, unsaturated fatty acids, vitamins, and other nutrients and low in fat, therefore, constituting a high-quality, low-calorie food [10]. In addition, the polysaccharides of *P. eryngii* demonstrate antioxidant, anti-tumor, antibacterial, anti-hyperlipidemic, and immunoregulatory activities [35]. The immunomodulating effects of *P. eryngii* polysaccharides have been investigated using different immunocompetent cells, such as monocytes and macrophages [36]. Macrophages are dynamic and heterogeneous cells. They can differentiate from peripheral monocytes by stimulation with various cytokines and growth factors. In infection, the activated monocytes in blood migrate to the inflamed tissues and differentiate into specific monocyte-derived macrophage populations that contribute to restoring tissue integrity and eliminating the pathogen [37]. Monocytes originate from bone marrow precursors and can differentiate to naïve macrophages, and upon activation, to a variety of subpopulations that can express more or less pro-inflammatory (M1 or classical activated macrophage) and/or anti-inflammatory (M2 or alternative activated macrophages) properties [38]. M1 macrophages express mediators of inflammation such as tumor necrosis factor α and β (TNF-α, TNF-β) and possess microbicidal and inflammatory functions. In contrast, M2 macrophages express several anti-inflammatory markers such as interleukin (IL)-10 or IL-13, playing an important role in the suppressive system of inflammation. Maintaining a balance of M1 and M2 macrophages could prevent from a development of an immune deficient disease, such as chronic inflammation, allergy, and cancer [39,40], all three related to ageing; thus, the M1/M2 balance maintenance could be critical for the elderly in particular.

Within the above context and in order to gain more insight into the mechanisms through which edible mushrooms exert their immunomodulatory effect and affect the balance of pro- and/or anti-inflammatory cytokines, the immunomodulating properties of FSs from *P. eryngii* were investigated in U937 (pro-monocytic, human myeloid leukemia cell line)-derived human macrophages and PBMCs, following in vitro fermentation of mushrooms by fecal inocula from healthy elderly volunteers (>60). Gene expression as well as levels of secreted pro- (TNF-α, IL-1β) and anti-inflammatory cytokines (IL-10, IL-1Rα) were assessed in the macrophages. In addition, CyTOF analysis was applied in selected volunteers’ PBMCs treated with their corresponding FSs under the same conditions in order to detect possible immunophenotypic changes on different immune cell populations.

## 2. Materials and Methods

### 2.1. Fungal Strains, Mushroom Cultivation, and Determination of Glucan Content

*P. eryngii* strain LGAM 216 was isolated in the frame of an ongoing investigation on the mushroom diversity in Greece. Pure cultures are maintained in the fungal Culture Collection of the Laboratory of General and Agricultural Microbiology (Agricultural University of Athens). The fungus was further cultivated in a substrate based on wheat straw and assessed with respect to mushroom production performance and glucans content as previously described [29].

### 2.2. Fecal Sample Collection and In Vitro Static Batch Culture Fermentations

Five fecal donors were apparently healthy subjects (>60 years), meeting the following inclusion criteria: (a) body mass index (BMI) <30 kg m^−2^, with no recent weight loss and extreme dietary behaviors; (b) no history of gastrointestinal disease, chronic constipation, chronic/acute diarrhea, autoimmune disease, coronary disease, and/or liver or kidney malfunction; (c) no consumption of antibiotics two months before the study; and (d) no consumption of probiotics, prebiotics, and/or dietary fiber supplements two weeks before the study, as previously described [34]. The study was conducted according to the guidelines laid down in the Declaration of Helsinki and under the approval of the Bioethics Committee of Harokopio University, Athens, Greece (62-03/07/2018). Written informed consent was obtained from all fecal donors prior to their inclusion in the study. Fecal sample collection, preparation of fecal inocula, and the in vitro static batch culture fermentation process were performed as previously described [34]. Briefly, stool samples were homogenized and diluted in phosphate-buffered saline (PBS) (0.1 M, pH 7.2) to produce a 20% (*w*/*v*) fecal slurry, which was subsequently added in the fermentation medium (10% *v*/*v*). Subsequently, 2% (*w*/*v*) of lyophilized *P. eryngii* powder was added in the fermentation medium (PE condition), and as a control, a second static batch culture in the absence of *P. eryngii* or any other additional carbon source apart from the fecal slurry was conducted in parallel (NC condition). Incubation was performed under anaerobic conditions at 37 °C. Samples were collected at 24 h, centrifuged at 10,000 × *g* for 45 min at 4 °C, and filtered with 0.25 μm Whatman^TM^ filters to obtain the fermentation supernatants (FS), which were stored at −80 °C until further use.

### 2.3. Cell Culture and Treatment

Human promonocytic leukemia U937 cells (ATCC^®^ CRL-1593.2™) were grown in RPMI medium (containing phenol red, nonessential amino acids, 1 mM sodium pyruvate, 2 mM L-glutamine, 1 g/L glucose, and 1.5 g/L sodium bicarbonate), supplemented with 10% fetal bovine serum (FBS; GIBCO, 10270) and 1% penicillin/streptomycin (Gibco-Life Technologies, Waltham, MA, USA), at 37 °C in a humidified incubator with 5% CO_2_. U937 cells were differentiated into macrophages as previously described [41]. Briefly, U937 cells were incubated in RPMI in the absence of FBS for 5 h in order to get synchronized. Subsequently 10^6^ cells were resuspended in 6-well plates in 2 mL medium (RPMI + 10% FBS + 1% penicillin/streptomycin), 100 ng/mL PMA (Phorbol 12-Myristate 13-Acetate; Tocris Biotechne, 1201) was added, and cell cultures were incubated at 37 °C in a humidified incubator with 5% CO_2_ for 48 h. Approximately 70% of these cells became adherent after treatment and show macrophage phenotype. Cells were then washed twice with 2 mL phosphate-buffered saline (PBS 1×, pH 7.2; GIBCO, 70013-016) and 2 mL of the culture medium was added to proceed to cell treatments.

U937 promonocytes and U937-derived macrophages were treated with 1% *v*/*v* fermentation supernatants (FSs of NC and PE fermentations) for 6 or 24 h. A negative control (RPMI; cell culture medium with no additional treatment) was included, whereas *E. coli*-derived lipopolysaccharide (LPS, 100 ng/mL) was used as positive control in macrophages. At the end of the incubation time, cell culture supernatants were collected, and cells were washed twice with 2 mL cold 1× PBS, then 0.5 mL TRIzol^®^ Reagent (Ambion by Life Technologies, Carlsbad, CA, USA, 15596018) was added and the resulting cell suspension was stored at −20 °C until further use.

Peripheral blood mononuclear cells (PBMCs) were isolated from whole blood immediately after collection of the blood sample. The cells were separated using Lymphoprep™ (STEMCELL Technologies, Oslo, Norway) density gradient medium. Briefly, 10 mL of collected blood was diluted with 10 mL PBS 1× and placed on 10 mL Lymphoprep™. Samples were then centrifuged at 2000 rpm for 25 min, and the middle layer containing the lymphocytes was transferred in 5 mL PBS 1×. After centrifugation at 2000 rpm for 15 min, the cell pellet was additionally washed twice with 5 mL PBS 1×, and cells were stored in freezing medium containing 50% FBS, 40% RPMI, and 10% dimethyl sulfoxide (DMSO; Sigma-Aldrich, Darmstadt, Germany) at −80 °C until further use. Before cell culture for CyTOF analysis, PBMCs were resuspended in cell culture medium (RPMI + 10% FBS + 1% penicillin/streptomycin) and incubated overnight at 37 °C in a humidified incubator with 5% CO_2_. The next day, no adherent cells were collected and resuspended in 6-well plates in 2 mL medium (2 × 10^6^ cells per well). Cells from each volunteer were treated with 1% *v*/*v* supernatants (NC and PE) collected from the in vitro fermentation performed by the same volunteer’s fecal sample. After 24 h of incubation, cells were washed twice with 1× PBS and resuspended in absolute RPMI, before proceeding to the cell staining protocol for Maxpar Direct Immune Profiling Assay.

### 2.4. Quantification of Cytokine Gene Expression in FS-Treated Macrophages

Total RNA was isolated from the cell suspensions mentioned in Section 2.3 following the TRIzol^®^ Reagent protocol for RNA extraction by Chomczynski (1993) [42] and RNA concentration in the resulting isolates was determined by BioPhotometer plus (Eppendorf Austria GmbH, Wien, Austria).

Complementary DNA (cDNA) was synthesized from 1 μg RNA extract diluted in 10 μL sterile ddH_2_O. Following the addition of 1 μL dNTP mix containing 10 mM dATP, 10 mM dTTP, 10 mM dGTP, and 10 mM dCTP (dNTP set, Thermoscientific, Thermo Fisher Scientific Baltics UAB, R0181) and 1 μL oligo(dT) 12–18 primer (Invitrogen by Thermo Fisher Scientific, Carlsbad, CA, USA, 18418012), RNA samples were incubated at 65 °C for 5 min and then on ice for 3 min. Subsequently, 4 μL 5× First-Strand Buffer (Invitrogen, P/N y02321), 2 μL 0.1 M DTT (Invitrogen, P/N Y00147), and 1 μL RNaseOUT (Invitrogen by Life Technologies, 10777-019) were added in the mix; after 2 min of incubation at 37 °C, 1 μL M-MLV reverse transcriptase (Invitrogen by Thermo Fisher Scientific, Carlsbad, CA, USA, 28025-013) was also added, and the final mix was incubated at 37 °C for 1 h. Samples were then transferred at 70 °C for 15 min and used immediately or stored at −20 °C until further use.

Real-time quantitative PCR was performed in order to quantify the gene expression of selected inflammation-related cytokines. Of the synthesized cDNA, 8 μL was mixed with 10 μL Luna^®^ Universal qPCR Master Mix (New England BioLabs Inc., Ipswich, MA, USA, M30003X) and 2 μL of primer pairs for the glyceraldehyde 3-phosphate dehydrogenase (*GAPDH*; Hs.PT.39a.22214836, Integrated DNA Technologies, Coralville, IA, USA) gene, interleukin-1β (*IL-1β*; Hs.PT.58.1518186, Integrated DNA Technologies), interleukin-1RN (*IL-1RN*, Hs.PT.58.4381999, Integrated DNA Technologies), interleukin-10 (*IL-10*; Hs.PT.58.2807216, Integrated DNA Technologies), or tumor necrosis factor (*TNF*; Hs.PT.5845380900, Integrated DNA Technologies) in a 20 μL reaction volume. Primer sets are shown in Table 1 and were tested by dilution series of cDNA from each cell type in order to analyze the PCR’s efficiency and determine the optimal cDNA dilution per set. The real-time instrument (CFX Connect Real-Time PCR Detection System; Bio-Rad Laboratories, Inc., Hercules, CA, USA) was programmed with the recommended thermocycling protocol, i.e., 60 s at 95 °C for initial denaturation followed by 42 cycles of 15 s at 95 °C, 30 s at 60 °C and plate read, and 10 additional seconds at 60 °C before data collection.

The housekeeping gene of *GAPDH* was chosen for normalization as it is stably expressed in both U937 promonocytes and macrophages. The relative mRNA expression was exhibited as fold change relative to the value of the negative control (untreated cells—RPMI condition) and calculated as ΔΔCt = 2^(ΔCt_GAPDH_ − ΔCt_sample_). All experiments were performed in duplicate, and the results were confirmed by a second independent experiment.

### 2.5. Quantification of Cytokine Secretion by FS-Treated Macrophages

Secretion of the cytokines IL-1β, IL-1Ra, IL-10, and TNF-α in U937-derived macrophages was measured in their cell culture supernatants via enzyme-linked immunosorbent assay (ELISA) following the manufacturer’s instructions and expressed as pg/mL. The levels of cytokines were obtained by comparison with the standard curve of each kit, which was generated from standards supplied by the manufacturer. Therefore, samples were diluted to the appropriate extent in order to provide a detectable measurement that would fit in the standard curve. All cell supernatants were centrifuged at 1000× *g* for 10 min right before the assay to remove particulates and aggregates. IL-1β was measured in 2× diluted supernatant samples (Human IL-1β ELISA kit, Cat. No: 850.006.192; Diaclone, Besançon, France), TNF-α was measured in 500× diluted supernatant samples (Human TNF-α ELISA kit, Cat. No: 950.060.192; Diaclone, France), IL-10 was measured in 10× diluted supernatant samples (Human IL-10 ELISA kit, Cat. No: 950.060.192; Diaclone, France), and IL-1Ra was measured in 70× diluted supernatant samples (BIOSOURCE IL-1ra Cytoscreen kit; KAC1181; BioSource Europe S.A., Nivelles, Belgium). All plates were read using a Universal Microplate Reader (Tecan Austria GmbH, Salzburg, Austria) with 450 nm as the primary wavelength and 620 nm as the reference wavelength.

### 2.6. CyTOF

After treatment with FS from NC or PE condition, PBMCs were washed with RPMI and stained for extracellular targets using the Maxpar Direct Immunophenotyping Assay [43] (Fluidigm, South San Francisco, CA, USA) according to the manufacturer’s instructions. Briefly, treated PBMCs were washed with CSB (Cell Staining Buffer), followed by a blocking step with anti-CD16/CD32 and staining with surface antibodies. Stained cells were then washed once with CSB and fixed with 1.6% methanol-free formaldehyde solution. Finally, stained-fixed cells were stained with the 191Ir/193Ir DNA intercalator overnight. The following day, cells were washed twice with CSB buffer and once with Cell Acquisition Solution (CAS) and resuspended with EQ normalization beads immediately before acquisition on a Helios™ system (Fluidigm, South San Francisco, CA, USA). Acquisition rate was maintained at a rate of ≈300–400 events/s. FCS files are available from Flow Repository (FR-FCM-Z52J).

### 2.7. Statistical Analysis

Results of cytokine secretion and gene expression were articulated by mean and standard deviations, and statistical analysis was conducted using IBM^®^ SPSS^®^ Statistics software (Version 23, Armonk, NY, USA). All groups passed the Shapiro–Wilk test for normality (*p* > 0.05; Sigmaplot 14.0); therefore, the results were analyzed by the parametric method of paired samples *t*-test, and the significance level was *p* ≤ 0.05 for all the calculations.

Regarding CyTOF, prior to analysis, data normalization was performed using Passport beads (Fluidigm method) with CyTOF software (version 10.7.1014). Then, a data cleanup step was performed using FlowJo™ v10.8 Software (BD Life Sciences, Franklin Lakes, NJ, USA) to gate for Gaussian parameters (residual, center, offset and width) and select live singlet DNA+ events. The dimensionality reduction algorithm viSNE was performed in the cloud-based analysis platform Cytobank [44]. To further analyze our data, we used the automatic workflow of the Maxpar Pathsetter™ software (Fluidigm method). Maxpar Pathsetter is an automated software that analyses data from FCS 3.0 files and applies workflow that eliminates debris, aggregates dead cells and normalization beads, and produces a report for 37 immune cell populations in peripheral blood based on predefined gates and definitions (Table S1). The Mann–Whitney test was performed using GraphPad Prism (version 9.2.0 for Windows, GraphPad Software, San Diego, CA, USA).

## 3. Results

### 3.1. Cytokine Expression and Secretion by U937-Derived Macrophages Treated with FSs

The potentially differential effect of FSs (from both NC and PE conditions) administration to inflammation-related cytokines at the levels of gene expression and protein secretion was investigated in U937-derived macrophages. Two pro-inflammatory (IL-1β and TNF-α) and two anti-inflammatory cytokines (IL-10 and IL-1Ra) were selected to be examined on the basis of previous preliminary experiments (data not shown). Gene expression of these cytokines of interest in macrophages treated with FSs and the protein secreted in the supernatants of FS-treated macrophages were measured by RT-qPCR and ELISA, respectively, at 6 and 24 h of treatment.

#### 3.1.1. TNF-α Gene Expression and Protein Secretion

**Figure 1:**
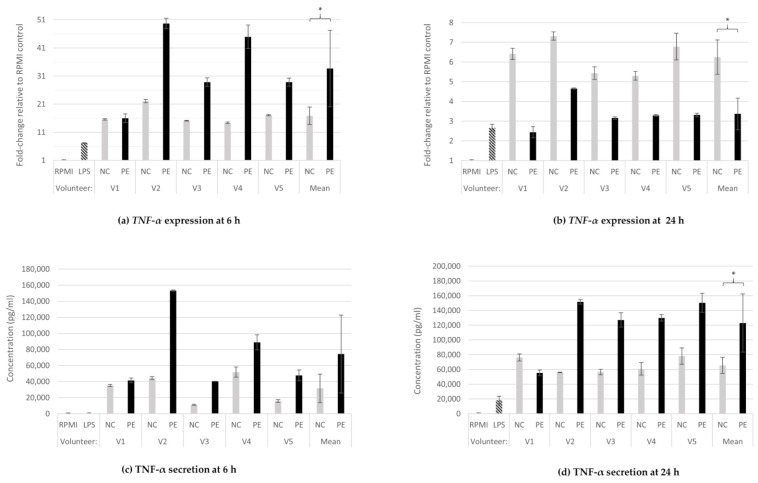
TNF-α gene expression and protein release. Relative expression of *TNF-α* at 6 h (**a**) and 24 h (**b**) of treatment of U937-derived macrophages with FSs, and TNF-α concentration in supernatants of the same cells at 6 h (**c**) and 24 h (**d**). Gene expression is exhibited as fold-change of the value in the untreated cells (RPMI) and relative towards *GAPDH* expression (ΔΔCt) for all samples (**a**,**b**). The concentration of the cytokine is expressed in pg/mL of the cell culture supernatants (**c**,**d**). Data shown are the means and standard deviation (SD bars) from two technical measurements. TNF-α concentration in RPMI at 6 and 24 h and LPS at 6 h were below detection levels. Asterisks indicate statistical significance as shown by paired-samples *t*-test, with * *p* < 0.05. RPMI: baseline, absence of treatment; LPS: treatment with 100 ng/mL lipopolysaccharide, positive control; NC: treatment with FSs from fermentation in the absence of additional carbon source; PE: treatment with FSs from fermentation in the presence of lyophilized mushroom powder of *P. eryngii*.

At 6 h of incubation with FSs, *TNF-α* expression was upregulated in all FSs treatments as well as in the positive control (LPS), in comparison to baseline expression of untreated cells (RPMI). Treatment of cells with FSs from PE condition led to significantly higher expression of *TNF-α* (34-fold increase) compared to NC FSs (17-fold increase) (Figure 1a; *p* = 0.038). However, at 24 h, the aforementioned expression profile of PE- versus NC-treated macrophages was inverted. PE treatments significantly decreased (*p* = 0.001) *TNF-α* expression in comparison to NC treatments, consistently in all five donors, with the mean expression being sixfold higher than RPMI in NC and three times higher in PE (Figure 1b). The upregulation in all FS treatments in comparison to baseline was still present at 24 h, but the effect was less prominent when compared to the relative fold-change of expression measured at 6 h. These effects were confirmed by a second independent experiment (Figure S1a).

TNF-α protein secretion levels increased in cell culture supernatants of FS-treated cells compared to baseline at both 6 (Figure 1c) and 24 h (Figure 1d). PE supernatants tended to have higher TNF-α concentrations than their NC counterparts already at 6 h of treatment (RPMI and LPS were below detection levels, NC 31,611 pg/mL, PE 74,949 pg/mL; *p* = 0.070); protein secretion continued to increase, and this difference became statistically significant at 24 h (RPMI: below detection levels, LPS: 18,316 pg/mL, NC: 65,497 pg/mL, PE: 122,864 pg/mL; *p* = 0.047).

#### 3.1.2. IL-10 Gene Expression and Protein Secretion

**Figure 2:**
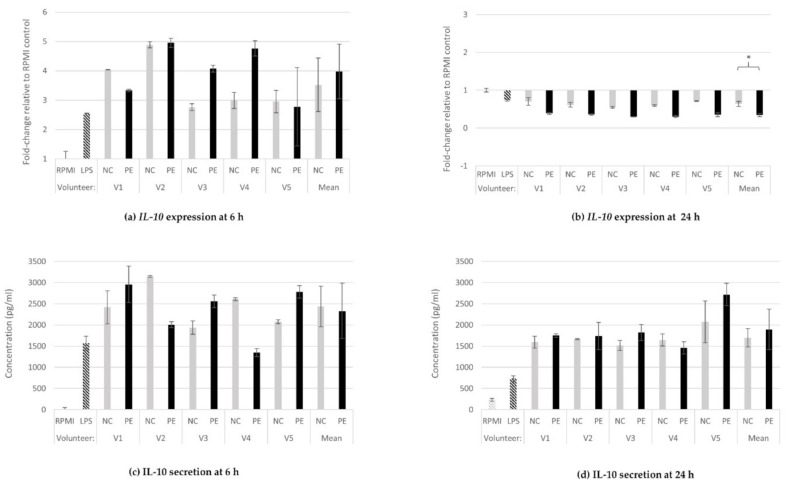
IL-10 gene expression and protein release. Relative gene expression of *IL-10* at 6 h (**a**) and 24 h (**b**) of treatment of U937-derived macrophages with FSs, and IL-10 concentration in supernatants of the same cells at 6 h (**c**) and 24 h (**d**). Gene expression is exhibited as fold-change of the value in the untreated cells (RPMI) and relative towards *GAPDH* expression (ΔΔCt) for all samples (**a**,**b**). The concentration of the cytokine is expressed in pg/mL of the cell culture supernatants (**c**,**d**). Data shown are the means and standard deviation (SD bars) from two technical measurements. IL-10 concentration in RPMI at 6 h was below detection levels. Asterisks indicate statistical significance as shown by paired-samples *t*-test, with * *p* < 0.05. RPMI: baseline, absence of treatment; LPS: treatment with 100 ng/mL lipopolysaccharide, positive control; NC: treatment with FSs from fermentation in the absence of additional carbon source; PE: treatment with FSs from fermentation in the presence of lyophilized mushroom powder of *P. eryngii*.

At 6 h of incubation with FSs, relative expression of *IL-10* was upregulated in all treatments, including the positive control (LPS; 2.6-fold increase), in comparison to baseline expression in untreated cells (RPMI). However, the degree of overexpression varied considerably depending on the volunteer, and no significant difference was detected in the expression level between the NC- (3.5-fold increase) and PE-treated (fourfold increase) samples overall (*p* = 0.386; Figure 2a). At 24 h, on the contrary, *IL-10* expression was downregulated below baseline expression levels in both NC (0.6-fold change) and PE (0.3-fold change) as well as in the LPS condition (0.7-fold change), with the effect in the PE-treated macrophages being significantly stronger than in their NC-treated counterparts (*p* = 0.0001; Figure 2b). These effects were confirmed by a second independent experiment (Figure S1b).

Regarding the cytokine’s secretion levels, IL-10 concentration increased in all cell culture supernatants derived from LPS- and FS-treated cells compared to baseline (RPMI) at both 6 (RPMI: below detection levels, LPS: 1571 pg/mL, NC: 2439 pg/mL. PE: 2331 pg/mL; Figure 2c) and 24 h (RPMI: 230 pg/mL, LPS: 734 pg/mL, NC: 1699 pg/mL, PE: 1898 pg/mL; Figure 2d). No significant differences were observed between the means of IL-10 levels in NC- and PE-treated macrophages.

#### 3.1.3. IL-1β Gene Expression and Protein Secretion

**Figure 3:**
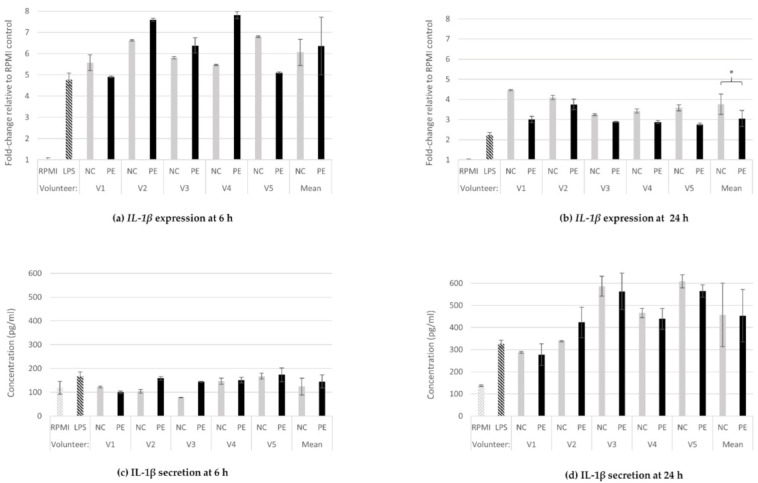
IL-1β gene expression and protein release. Relative gene expression of *IL-1β* at 6 h (**a**) and 24 h (**b**) of treatment of U937-derived macrophages with FSs, and IL-1β concentration in supernatants of the same cells at 6 h (**c**) and 24 h (**d**). Gene expression is exhibited as fold-change of the value of the value in the untreated cells (RPMI) and relative towards *GAPDH* expression (ΔΔCt) for all samples (**a**,**b**). The concentration of the cytokine is expressed in pg/mL of the cell culture supernatants (**c**,**d**). Data shown are the means and standard deviation (SD bars) from two technical measurements. Asterisks indicate statistical significance as shown by paired-samples *t*-test, with * *p* < 0.05. RPMI: baseline, absence of treatment; LPS: treatment with 100 ng/mL lipopolysaccharide, positive control; NC: treatment with FSs from fermentation in the absence of additional carbon source; PE: treatment with FSs from fermentation in the presence of lyophilized mushroom powder of *P. eryngii*.

The relative *IL-1β* expression at 6 (Figure 3a) and 24 h (Figure 3b) of incubation with FSs was increased in all FS treatments as well as in the positive control (LPS). No significant difference between NC- (6.1-fold increase) and PE-treated cells (6.4-fold increase) was observed at 6 h of incubation; however, at 24 h, PE treatments led to significant decrease in the expression of this pro-inflammatory cytokine in comparison to NC (3.1 vs. 3.8- fold change, respectively (*p* = 0.027), as confirmed by a second independent experiment (Figure S1c).

There were no differences, compared to baseline (RPMI), regarding IL-1b protein secretion levels between PE- and NC-treated cells (RPMI: 119 pg/mL, LPS: 168 pg/mL, NC: 124 pg/mL, PE: 145 pg/mL; Figure 3c); at 24 h, LPS (326 pg/mL). Both NC (457 pg/mL) and PE (453 pg/mL) treatments from all volunteers significantly increased IL-1β levels, but no significant difference between NC and PE means was observed (Figure 3d).

#### 3.1.4. IL-1Ra Gene Expression and Protein Release

**Figure 4:**
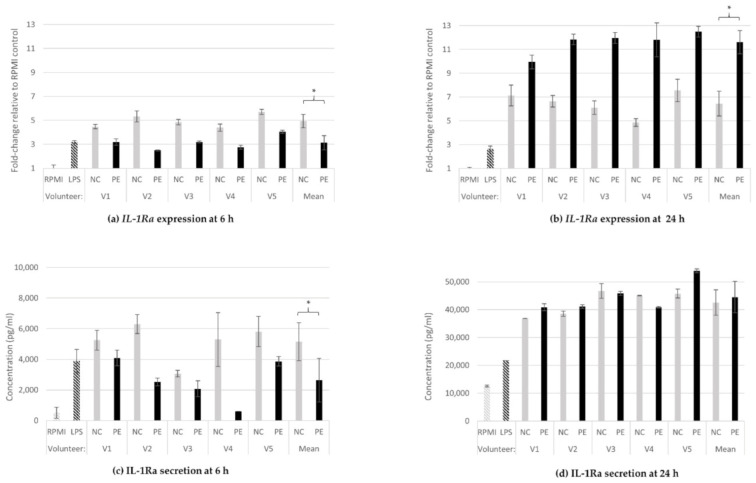
IL-1Ra gene expression and protein release. Relative gene expression of *IL-1Ra* at 6 h (**a**) and 24 h (**b**) of treatment of U937-derived macrophages with FSs, and IL-1Ra concentration in supernatants of the same cells at 6 h (**c**) and 24 h (**d**). Gene expression is exhibited as fold-change of the value in the untreated cells (RPMI) and relative towards *GAPDH* expression (ΔΔCt) for all samples (**a**,**b**). The concentration of the cytokine is expressed in pg/mL of the cell culture supernatants (**c**,**d**). Data shown are the means and standard deviation (SD bars) from two technical measurements. Asterisks indicate statistical significance as shown by paired-samples *t*-test, with * *p* < 0.05. RPMI: baseline, absence of treatment; LPS: treatment with 100 ng/mL lipopolysaccharide, positive control; NC: treatment with FSs from fermentation in the absence of additional carbon source; PE: treatment with FSs from fermentation in the presence of lyophilized mushroom powder of *P. eryngii*.

*IL-1Ra* expression was upregulated in all FS treatments as well as in the positive control (LPS) at both 6 (Figure 4a) and 24 h (Figure 4b), in comparison to baseline expression of untreated cells (RPMI). Interestingly, the comparison between the means of NC- and PE-treated cells revealed a time-dependent inversion of their relative expression profile. Namely, at 6 h, the NC treatment led to significantly higher expression (4.9-fold increase) than PE (3.1-fold increase) for all volunteers (*p* = 0.002; Figure 4a), while at 24 h, PE-treated macrophages showed significantly higher expression (11.6-fold increase) compared to NC-treated ones (6.5-fold increase, *p* = 0.002; Figure 4b). These effects were confirmed by a second independent experiment (Figure S1d).

The aforementioned inversion of the effect in *IL-1Ra* expression was somewhat reflected at the protein secretion levels at the same time points. Treatment of cells with PE FSs for 6 h significantly decreased the levels of IL-1Ra secretion compared to both LPS and NC (RPMI: 517 pg/mL, LPS: 3893 pg/mL, NC: 5143 pg/mL, PE: 2634 pg/mL; *p* = 0.027 for mean NC-PE mean comparison; Figure 4c), while at 24 h, protein secretion levels were significantly increased in both NC- and PE-treated cell supernatants, and no statistically significant difference was observed between the two conditions (RPMI: 12,562 pg/mL, LPS: 21,739 pg/mL, NC: 42,586 pg/mL, PE: 44,510 pg/mL; Figure 4d).

### 3.2. CyTOF Pilot Study

**Figure 5:**
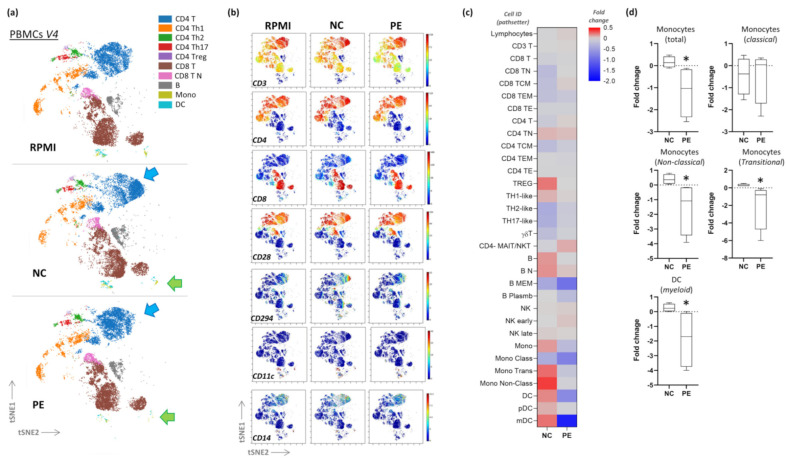
Phenotyping of treated PBMCs with NC/PE fermentation supernatants (FSs) with mass cytometry. (**a**) viSNE maps presenting the similarities of identified cell types (cell definitions are based on automatic gating performed in Cytobank—each dot represents a single cell) in PBMCs of V4 after different treatments (RPMI, NC, and PE) and (**b**) viSNE maps presenting the expression of markers CD3, CD4, CD8, CD294, CD28, CD11c, and CD14 on this analysis. (**c**) Heatmap showing fold changes in abundance of different cell types identified with the Pathsetter analysis in treated PBMCs from all volunteers and (**d**) boxplots for monocytes and myeloid DCs. RPMI: baseline, absence of treatment; NC: treatment with FSs from fermentation in the absence of additional carbon source; PE: treatment with FSs from fermentation in the presence of lyophilized mushroom powder of *P. eryngii*. Asterisks indicate statistical significance as shown by Mann–Whitney test, with * *p* < 0.05.

CyTOF analysis was applied in selected volunteers’ PBMCs (V1, V2, V3, V4) treated with their corresponding FSs under the same conditions. FS-treated PBMCs were analyzed for immunophenotypic changes on different immune cell populations by utilizing mass cytometry (CyTOF technology) that allows the resolution of many different parameters simultaneously at the single cell level. We used the 30-marker panel Maxpar Direct Immunophenotyping Assay that allows analysis of many immune cell subpopulations in peripheral blood. Initially, we performed visualization of t-distributed stochastic neighbor embedding (viSNE) to compare cell population according to treatments with different FSs. Exploratory analysis of the PBMCs of the four volunteers revealed marked differences in different cellular identities projected on the viSNE map (Figure S2). A representative illustration of volunteer 4 (V4) is shown in Figure 5a,b. In the NC condition, a cluster of activated T helper type 2 (Th2) cells (CD3 + CD4 + CD8-) expressing CD28 and CD294 was observed. This cluster was not present in baseline RPMI- and PE-treated PBMCs. Moreover, this exploratory analysis also revealed differences in less abundant cells of the myeloid lineage (non-T/B cells) that expressed CD11c and CD14; these clusters appeared more abundant in the NC condition, while they were rather scarce in the PE condition. These data from all volunteers were further analyzed with the automatic workflow Pathsetter (Tables S1 and S2). Overall, compared to baseline RPMI condition, NC-treated PBMCs exhibited small fold increases in certain populations of monocytes and dendritic cells as opposed to the PE condition. Interestingly, significant fold decrease in the percentages of non-classical and transitional monocytes (characterized by the expression of CD16 and CD14), as well as of myeloid dendritic cells (DCs), was observed in the PE-treated PBMCs, as shown in Figure 5c,d for V4.

## 4. Discussion

In this study, we investigated the immunomodulating effects of *P. eryngii* mushrooms, previously selected due to their high β-glucan content, strong lactogenic effect, and potent geno-protective properties, following in vitro fermentation by fecal inocula from healthy elderly volunteers (>60 years old) [29,34]. The immunomodulating properties of the fermentation supernatants (FSs) were investigated in U937-derived human macrophages and PBMCs. Therefore, the study assessed the indirect effects of *P.eryngii* on two critical branches of the immune system. The FSs used include all metabolites produced in the course of the *P. eryngii* in vitro fermentation, SFCAs for the most part [29], reflecting changes in the fecal microbiota composition of healthy elderly volunteers.

Macrophages are a major component of the innate immune system and important source of cytokine secretion following infection. Among the metabolites derived from intestinal microbial fermentation of indigestible food components, short-chain fatty acids (SCFAs) regulate the function of intestinal-associated immune cells. For instance, butyrate has an anti-inflammatory effect by inhibiting the recruitment and pro-inflammatory activity of neutrophils, macrophages, dendritic cells, and effector T cells, and by increasing the number and activity of regulatory T cells [45]. SCFAs can also regulate the differentiation of T cells and B cells and the antigen-specific adaptive immunity mediated by them [46]. Under homeostatic conditions, macrophages are one of the most abundant cell types of the intestinal immune system, differentiated mainly responding to local signaling. Their function is largely regulated by the intestinal microflora and, as a result, they are more abundant in the colon than in the small intestine, where they can be found in close association with epithelial cells. Impaired sub-epithelial localization contributes to the loss of intestinal barrier integrity seen in inflammatory bowel diseases such as ulcerative colitis and Crohn’s disease. In general, macrophages seem to orchestrate epithelial cell-microbiota interactions for maintenance of colon homeostasis [47].

In the context of preliminary experiments, changes in the gene expression of TNF-α, IL-1β, IL-6, IL-8, IL-1Rα, and IL-10 were assessed in U937-derived human macrophages. The main finding of this initial screening was that the expression of all the cytokines examined was altered upon the effect of fermentation supernatants (1% on the RPMI medium) consistently in all cases compared to baseline expression, which indicates the strong immunoregulatory potential of the products of the fecal microbiota metabolism (data not shown). Furthermore, in vitro fermentation in the presence of *P. eryngii* lyophilized mushroom powder (PE condition) differentiated the response of cytokines TNF-α, IL-1β (pro-inflammatory), and IL-1Rα, IL-10 (anti-inflammatory) only. Hence, we proceeded with those four cytokines and thoroughly investigated their changes at both transcriptional (gene expression) and translational level (protein secretion) at 6 and 24 h of FSs treatment.

TNF-α is a pro-inflammatory cytokine that plays a central role in inflammatory and immune processes. It is naturally produced by activated macrophages and monocytes, and has pleiotropic effects on normal and malignant cells as it regulates the inflammatory response and inhibits carcinogenesis [48]. In our study, TNF-α protein secretion assessed at 6 h was in compliance with the gene expression trend as shown in Figure 1a,c; at the transcriptional level, PE FS treatment significantly increased the *TNF-α* expression levels relative to the control NC (no additional carbon source) FSs. At the translational level, the secreted protein increased in PE relative to NC, although the increase was not statistically significant. At 24 h, the *TNF-α* expression levels in PE condition were significantly decreased relative to NC, while the levels of the secreted protein continued to increase in the PE condition relative to NC. TNF-α was the only cytokine for which a statistically significant difference at protein secretion was observed between NC and PE conditions (Figure 1d, Figure 2d, Figure 3d and Figure 4d). The downregulation of the *TNF-α* expression in PE condition at 24 h is most likely due to the feedback loop involved in the regulation of TNF-α, where high levels of this protein bind to tumor necrosis factor receptor-2 (TNFR2) receptors and induce *IL-10* expression, which in turn suppresses *TNF-α* expression [49]. Indeed, already at 6 h of treatment, *TNF-α* expression was significantly higher in PE than in NC and was accompanied by increased protein levels until 24 h (Figure 1a–d). IL-10 levels remained high throughout the assessment, from 6 to 24 h (Figure 2c,d); this supports the involvement of IL-10-mediated feedback inhibition on *TNF-*α transcription in macrophages [49]. It is important that the production of pro-inflammatory cytokines is kept under control to avoid excess inflammation and tissue damage, as well as to allow the eventual resolution of inflammation. This occurs by both the induction of direct intracellular negative feedback mechanisms and by the action of anti-inflammatory cytokines such as IL-10 and IL-1 receptor antagonist (IL-1Ra). IL-10 induction often occurs in conjunction with pro-inflammatory cytokines, although IL-10-inducing pathways may negatively regulate them [50]. The high TNF-α levels at 24 h after administration of fermentation supernatants in the presence of *P. eryngii* (Figure 1d) are consistent with recent studies, suggesting that in vitro administration of mycelial extracts of *P. ostreatus* induces the secretion of pro-inflammatory cytokines such as TNF-α in RAW 264.7 macrophages [51]. Moreover, when *P. eryngii* polysaccharides are administered to mice, they significantly inhibit tumor growth and enhance the immune function of these mice by increasing serum levels of the cytokines IL-2, TNF-α, and interferon-c (IFN-c), as well as natural killer cells (NKs) and cytotoxic T cells (CTLs) [52].

IL-10 is an anti-inflammatory cytokine that plays a key role in regulating inflammation and autoimmune pathology. Elevated IL-10 levels can interfere with the host’s response to microbial infections, repair tissue damage, and lead to hemodynamic disturbances. In contrast, deficient IL-10 levels can lead to the development of autoimmune disease and oncogenesis [53]. IL-10 is widely expressed by many immune cells, and the regulation of its expression and production is complex at both the transcriptional and post-transcriptional levels, depending on the biological environment, type of stimulus, tissue specificity, and individual’s genetic background [53,54]. It is extremely interesting that in our study, *IL-10* was the only cytokine gene downregulated in relation to baseline expression 24 h after administration of fermentation supernatants (NC and PE) and LPS. In the presence of *P. eryngii*, the gene expression levels decreased even more compared to NC (Figure 2b). At the translational level, the secreted protein remained high, while no statistically significant difference between the NC and PE conditions was observed (Figure 2c,d). Since IL-10 is a potent immunosuppressive cytokine, its activity must be tightly regulated, and it has been shown that IL-10 itself can drive its own expression in an autocrine manner in many immune effector types through activation of the signal transducer and activator of transcription 3 (STAT3) [55]. Stimulation of macrophages by Toll-like receptor (TLR) agonists results in the secretion of TNF-α, IL-1, IL-6, and IL-12, and the production of these cytokines is controlled by multiple feedback pathways. Macrophages also produce IL-10, which acts by inhibiting the production of pro-inflammatory cytokines through a Janus kinase (JAK)/STAT3-dependent signaling pathway [53,56]. The fact that in the present study, induction of IL-10 and TNF-α protein secretion levels was observed, at both 6 and 24 h after administration of PE and NC FSs, accompanied by significant downregulation of *IL-10* expression and reduction of *TNF-*α expression only in the PE condition (Figure 1 and Figure 2) indicates the importance of the tight regulation of *IL-10* transcription, and suggests the function of an IL-10 mediated anti-inflammatory feedback mechanism, which is even more effective in the case of PE FS administration. Overall, it seems that the supernatants of the in vitro fermentation carried out in the presence of *P. eryngii* are more effective in counterbalancing the increase in TNF-α by a simultaneous, tightly regulated synthesis of IL-10 in macrophages. This finding, in compliance with previous observations [57,58], highlights the importance of TNF-α/IL-10 balance for immune homeostasis maintenance. Given the fact that, as it has recently been shown, the therapeutic efficacy of anti-TNF agents administered against intestinal inflammation is highly dependent on IL-10 signaling in macrophages [59], further investigation of the effect of *P. eryngii* at the JAK/STAT3 signaling pathway is of great importance.

Similar to *TNF-α*, the expression of the pro-inflammatory cytokine *IL-1β* was induced relative to the positive LPS control after administration of fermentation supernatants (NC, PE) (Figure 3a,b). While at the translational level, no significant difference was observed between NC and PE (Figure 3c,d), in the presence of *P. eryngii*, the expression was significantly decreased at 24 h, an observation connected to the results regarding the IL-1 receptor antagonist (IL1Ra) (Figure 4a–d); contrary to *IL-1β*, in the presence of *P. eryngii*, *IL-1Ra* expression was significantly increased at 24 h, accompanied by increased protein secretion (Figure 4b). IL-1α and IL-1β are proinflammatory cytokines that participate in the regulation of immune responses, inflammatory reactions, and hematopoiesis. The IL-1 ligands and receptors are primarily associated with acute and chronic inflammation, and their effects are more intense than other cytokines [60]. It is known that there are multiple levels of negative regulation within the IL-1 cytokine family and one the major negative regulators is IL-1 receptor antagonist alpha (IL-1Ra) [61,62]. Recombinant IL-1Ra (generic anakinra) is fully active in blocking the IL-1 receptor 1 (IL-1R1), and thus the activities of IL-1α and IL-1β, and is approved for the treatment of rheumatoid arthritis and several other diseases [62]. It is important that *P. eryngii* FSs significantly upregulated the *IL1-Ra* expression and IL-1Ra secretion to counterbalance the IL-1β pro-inflammatory effect. The connection between inflammation and colitis-associated intestinal cancer has been established, and signals originating from microbes and dysbiosis are key drivers of gastrointestinal tumorigenesis [63,64]. IL-1 is downstream of microbial sensing by epithelial cells or innate immunity cells and has emerged as a key component of tumor-promoting inflammation [61]; therefore, it is crucial to further identify and characterize the bioactive components of *P. eryngii* responsible for the anti-inflammatory effect observed.

It is worthwhile mentioning that, when in vitro fermentations were carried out in the presence of yeast β-glucan and β-glucan-enriched *P. eryngii* extract, the FSs did not affect cytokine expression compared to NC (data not shown). Therefore, the immunomodulatory effect of *P. eryngii* is likely due to a wider range of bioactive substances, most likely polysaccharides in addition to β-glucans, a result in agreement with recent findings [52,65].

In order to further investigate the effect of *P. eryngii* on PBMCs, FSs corresponding to four selected volunteers were analyzed for immunophenotypic changes on different immune cell populations by CyTOF. In the NC condition, a cluster of activated T helper (Th) cells (CD3 + CD4 + CD8-) expressing CD28 and CD294 was observed (Figure 5a,b). CD28 is the receptor for the B7 molecule of antigen-presenting cells, and CD294 (also known as Prostaglandin D2 receptor 2 (DP2) or CRTH2) is a chemoattractant receptor expressed by activated T helper type 2 (Th2) cells and mediates recruitment and activation of granulocytes and T cells at sites containing mast cells activated by invading allergens [66]. Interestingly, this cluster was not present in the baseline (RPMI) and PE condition (Figure 5a,b). Th2 cells are important in humoral immunity and defense against many allergic inflammatory diseases [67]. Moreover, this exploratory analysis also revealed differences in less abundant cells of the myeloid lineage (non-T/B cells) that expressed CD11c, also known as integrin alpha X, the most widely used defining marker for dendritic cells (DCs), and cluster of differentiation 14 (CD14), a monocyte/macrophage differentiation antigen on the surface of myeloid lineage, such as monocytes, macrophages, and dendritic cells (DCs). These clusters appeared more abundant in the NC condition and rather scarce in the PE condition. The myeloid lineage was not properly represented in our analysis, since a considerable fraction of these cells have probably been lost, as adherent, due to PBMCs’ isolation and culture conditions (Section 2.3). Nevertheless, despite this limitation, we further analyzed these data from all four volunteers with the automatic workflow Pathsetter (Tables S1 and S2). Compared to baseline RPMI condition, small fold increases in certain populations of monocytes and dendritic cells in NC-treated PBMCs were observed as opposed to the PE condition. Interestingly, in PE-treated PBMCs, a significant fold decrease in the percentages of non-classical and transitional monocytes (characterized by the expression of CD16 and CD14) as well as myeloid DCs was observed (Figure 5c,d). Overall, the findings of this pilot study provided evidence supporting consumption of *P. eryngii* mushrooms, as they could reverse activation of the immune system derived from certain fecal microbiota metabolites and help to maintain immune homeostasis not only in the intestinal environment but overall in the body.

In conclusion, the presence of *P. eryngii* in the in vitro fermentation process clearly affected immune response, as indicated by the altered gene expression and secretion levels of pro- (TNF-α, IL-1β) and anti-inflammatory cytokines (IL-10, IL-1Rα) in human macrophages. Furthermore, fermentation supernatants induced immunophenotypic changes in Th2 and myeloid lineage cells in PBMCs of healthy elderly volunteers. Our results provide evidence supporting a potent, beneficial immunomodulatory action for this edible mushroom in the elderly, which supports the perspective of its utilization in functional food production. Further studies encompassing more volunteers and holistic methodolical approaches (i.e., NGS-metataxonomics, NMR-metabolomics, cross-omics bioinformatics) are currently underway in order to validate these findings, correlate them with specific fermentation metabolites and fecal microbiota composition, and eventually determine the molecular mechanisms underlying these effects.

## Figures and Tables

**Table 1:** Primer sequences used in real-time qPCR for gene expression quantification.

Gene	RefSeqNumber	Sequence (5′ -> 3′)
*GAPDH*	NM_002046	Primer 1: 5′-ACATCGCTCAGACACCATG-3′ Primer 2: 5′-TGTAGTTGAGGTCAATGAAGGG-3′
*IL-1β*	NM_000576	Primer 1: 5′-CAGCCAATCTTCATTGCTCAAG-3′ Primer 2: 5′-GAACAAGTCATCCTCATTGCC-3′
*IL-1RN*	NM_173843	Primer 1: 5′-CTGTCCTGTGTCAAGTCTGG-3′ Primer 2: 5′- TTGTCCTGCTTTCTGTTCTCG-3′
*IL-10*	NM_000572	Primer 1: 5′-GCGCTGTCATCGATTTCTTC-3′ Primer 2: 5′-TCACTCATGGCTTTGTAGATGC-3′
*TNF*	NM_000594	Primer 1: 5′-TGCACTTTGGAGTGATCGG-3′ Primer 2: 5′-TCAGCTTGAGGGTTTGCTAC-3′

## Data Availability

CyTOF data, fcs files, are publicly available from Flow Repository (https://flowrepository.org/), FR-FCM-Z52J.

## Supplementary Materials

The following supporting information can be downloaded at https://www.mdpi.com/article/10.3390/jof8040329/s1. Figure S1. Cytokine expression: relative expression of *TNF-α, IL-1-, IL-1β,* and *IL-1Ra*, 6 h and 24 h after treatment of U937-derived macrophages with FSs. Figure S2. viSNE maps of all 30 markers of the MDIPA panel. Table S1. Pathsetter cell-type definitions. Table S2. Pathsetter analysis on Maxpar Direct Immunophenotyping Assay of treated PBMCs.
