# Supplementary material for: Immunomodulating Activity of Pleurotus eryngii Mushrooms Following Their In Vitro Fermentation by Human Fecal Microbiota
Source: J Fungi (Basel). 2022 Mar 22;8(4):329. doi: 10.3390/jof8040329 (PMC9028658; doi:10.3390/jof8040329)
Supplement: Supplementary file 1 [file jof-08-00329-s001.zip › Figure S1.pdf]

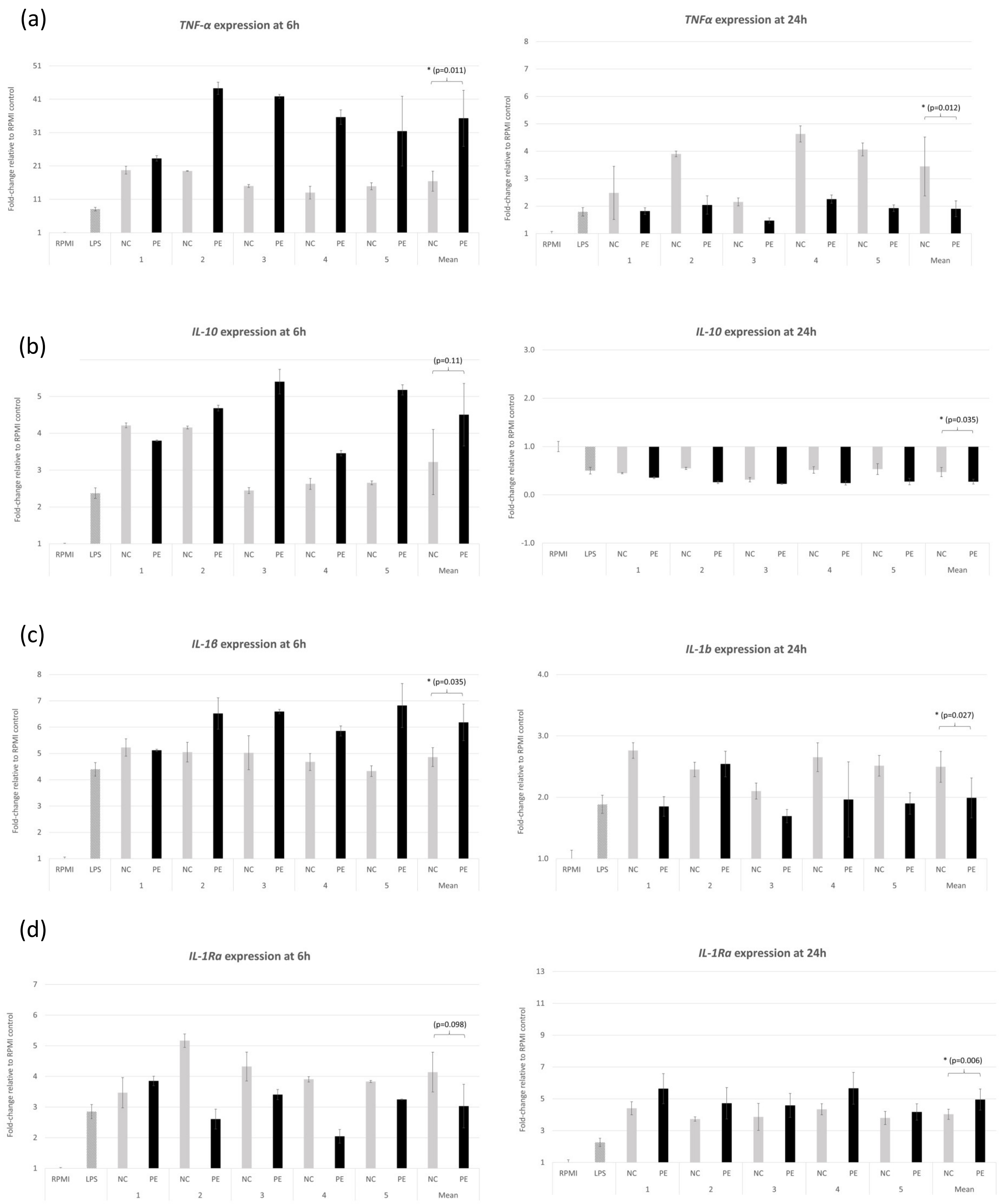

Figure S1. Cytokine expression - confirmation experiment. Relative gene expression of (a) *TNF-α*, (b) *IL-10*, (c) *IL-1β* and (d) *IL-1Ra* at 6 h and 24 h of treatment of U937-derived macrophages with FSs. Gene expression is exhibited as fold-change of the value in the untreated cells (RPMI) and relative towards *GAPDH* expression (ΔΔCt) for all samples. Data shown are the means and standard deviation (SD bars) from two technical measurements. Asterisks indicate statistical significance as shown by paired-samples t-test, with $p<0.05$. RPMI: Baseline, absence of treatment; LPS: Treatment with 100 ng/ml lipopolysaccharide, positive control; NC: Treatment with FSs from fermentation in the absence of additional carbon source; PE: Treatment with FSs from fermentation in the presence of lyophilized mushroom powder of *P. eryngii*.
