# Supplementary material for: Immunomodulating Activity of Pleurotus eryngii Mushrooms Following Their In Vitro Fermentation by Human Fecal Microbiota
Source: J Fungi (Basel). 2022 Mar 22;8(4):329. doi: 10.3390/jof8040329 (PMC9028658; doi:10.3390/jof8040329)
Supplement: Supplementary file 1 [file jof-08-00329-s001.zip › Figure S2.pdf]

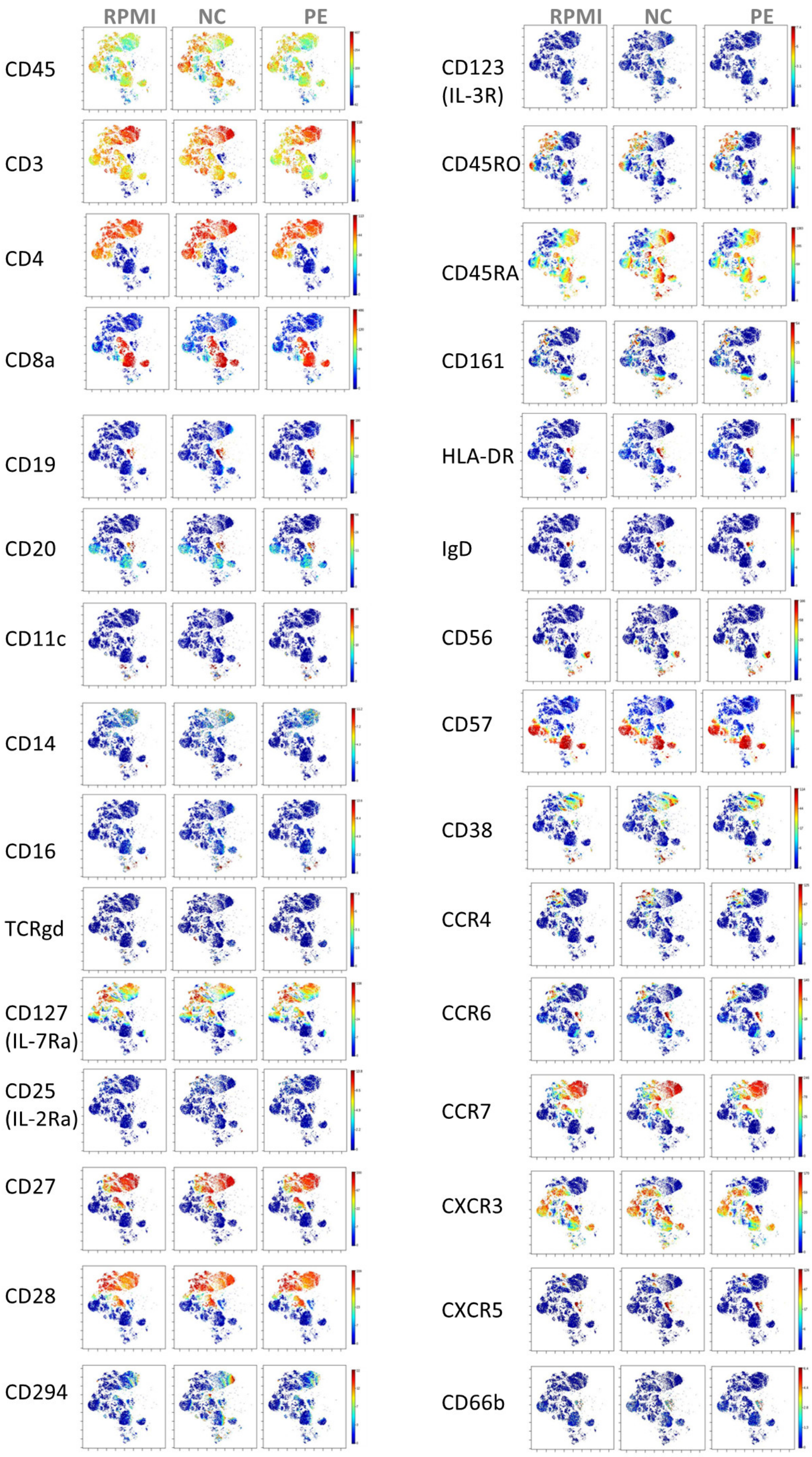

Figure S2. viSNE maps of all 30 markers of the MDIPA panel. Each map contains two dimensions (x) t-SNE1 and (y) t-SNE2 that project the structure of the data and phenotypic similarities of the identified cells for all conditions (RPMI, NC and PE-treated PBMCs) and as well as a third dimension (z) that colors the map (low-blue to high-red expression) based on the expression of each marker shown on the left of each plot. RPMI: Baseline, absence of treatment; NC: Treatment with FS in the absence of additional carbon source; PE: Treatment with FS in the presence of lyophilized mushroom powder of *P. eryngii*.
