# Supplementary material for: Immunomodulating Activity of Pleurotus eryngii Mushrooms Following Their In Vitro Fermentation by Human Fecal Microbiota
Source: J Fungi (Basel). 2022 Mar 22;8(4):329. doi: 10.3390/jof8040329 (PMC9028658; doi:10.3390/jof8040329)
Supplement: Supplementary file 1 [file jof-08-00329-s001.zip › Table S1.pdf]

Table S1. Pathsetter Cell type definitions. Cell type names, abbreviations used for Figure 1c and marker description according to the automatic analysis workflow Pathsetter (ref). This workflow also includes granulocytes that were not part our analysis.

	Cell type	Abbreviation	Pathsetter analysis - marker description - gating
1	*Lymphocytes*	**Lymphocytes**	CD3+, CD66b- CD19+, CD20+, CD45+, CD14-
2	*CD3 T Cells*	**CD3 T**	CD3+ CD66b- CD19-, CD20-, CD45+, CD14-
3	*CD8 T Cells*	**CD8 T**	CD3+ CD66b- CD19- CD8+ CD4- CD14- CD161- TCRgd- CD123- CD11c- CCR7+>- CD27+>-
4	*Naive*	**CD8 TN**	CCR7+, CD45RA+, CD45RO-
5	*Central memory*	**CD8 TCM**	CCR7+, CD45RA-, CD45RO+
6	*Effector memory*	**CD8 TEM**	CCR7-, CD45RA+, CD45RO-
8	*CD4 T Cells*	**CD4 T**	CD66b- CD3+ CD8- CD4+ CD14- TCRgd- CD11c- CCR7+>- CD27+>- CD45RA+>-
9	*Naive*	**CD4 TN**	CCR7+, CD45RA+, CD45RO-
10	*Central memory*	**CD4 TCM**	CCR7+, CD45RA-, CD45RO+
11	*Effector memory*	**CD4 TEM**	CCR7-, CD45RA+, CD45RO-
12	*Terminal effector*	**CD4 TE**	CCR7-, CD45RA-, CD45RO+
13	*Treg*	**TREG**	CD25+CCR4+ CD127-
14	*Th1-like*	**Th1-like**	CXCR3+ CCR6- CXCR5-
15	*Th2-like*	**Th2-like**	CXCR3- CCR6- CXCR5- CCR4+
16	*Th17-like*	**Th17-like**	CXCR3- CCR6+ CXCR5- CCR4+
17	*Gamma Delta T Cells*	**γδT**	CD66b- CD3+ CD8dim,- CD4- CD14- TCRgd dim,+
18	*MAIT & NKT CD4- Cells*	**MAIT/NKT CD4 T**	CD66b- CD3+ CD4- CD14- CD161 dim,+ TCRgd- CD28+ CD16- CD161+
19	*B Cells*	**B**	CD3- CD14- CD56- CD16 dim,- CD19+ CD20+ HLA-DR dim,+ CD27->+
20	*Naive*	**B N**	IgD+, CD27-
21	*Memory*	**B MEM**	IgD-, CD27+
22	*Plasmablasts*	**B Plamb**	CD38++ CD27+
23	*NK Cells*	**NK**	CD14- CD3- CD123- CD66b- CD45RA+ CD56dim,+
24	*Early NK*	**NK early**	CD16-
25	*Late NK*	**NK late**	CD16+
26	*Monocytes*	**Mono**	CD3- CD19- CD56- CD66b- HLA-DR+ CD11c+ CD14->+ CD38->+
27	*Classical*	**Mono Class**	CD16-, CD14++
28	*Transitional*	**Mono Trans**	CD16+, CD14++
29	*Non-classical*	**Mono Non-Class**	CD16++, CD14->+
30	*Dendritic Cells*	**DC**	CD3- CD19- CD14- CD20- CD66b- HLA-DR dim,+ CD11c- CD123 +
31	*pDC*	**pDC**	CD3- CD19- CD14- CD20- CD66b- HLA-DR dim,+ CD11c- CD123 +
32	*mDC*	**mDC**	CD3- CD19- CD14- CD20- HLA-DR dim,+ CD11c dim,+ CD123- CD16 dim,- CD38 dim,+ HLA-DR++
