# Supplementary material for: Immunomodulating Activity of Pleurotus eryngii Mushrooms Following Their In Vitro Fermentation by Human Fecal Microbiota
Source: J Fungi (Basel). 2022 Mar 22;8(4):329. doi: 10.3390/jof8040329 (PMC9028658; doi:10.3390/jof8040329)
Supplement: Supplementary file 1 [file jof-08-00329-s001.zip › Table S2.pdf]

Table S2. Pathsetter analysis on Maxpar Direct Immunophenotyping Assay of treated PBMCs. Raw data output percentages of the different cell types from the pathsetter cleaned fcs reports (top panel) for the different conditions (RPMI, NC and PE) of all volunteers and fold change calculations compared to baseline (RPMI) (bottom panel). RPMI: Baseline, absence of treatment; NC: Treatment with FS in the absence of additional carbon source; PE: Treatment with FS in the presence of lyophilized mushroom powder of *P. eryngii.*

Pathsetter analysis — Cells IDs

Sample	Condition	Lymph	CD3 T cells	CD8 T cells	Naïve	CM	EM	TE	CD4 T cells	Naïve	CM	EM	TE	TREG	TH1-like	TH2-lke	TH17-like
V4	0_RPMI	89,14	82,26	29,47	2,88	1,28	1,71	23,60	50,95	17,71	7,44	4,72	21,09	0,39	13,91	2,50	1,48
V3	0_RPMI	91,77	39,67	13,16	4,24	0,44	1,62	6,86	25,49	11,10	3,13	3,90	7,36	0,23	3,26	1,45	1,20
V1	0_RPMI	82,89	61,22	7,90	1,02	1,29	0,00	5,86	51,35	7,23	22,79	0,00	31,46	0,42	3,48	12,81	1,80
V2	0_RPMI	89,12	73,14	17,69	1,10	0,47	1,67	14,45	49,42	11,33	9,36	0,00	39,18	0,41	5,34	7,72	2,20
V4	1_NC	86,66	78,69	28,24	3,25	0,88	1,17	22,95	48,64	18,59	6,07	4,51	19,47	0,58	11,89	2,46	1,33
V3	1_NC	90,33	30,14	9,39	2,41	0,21	1,16	5,61	20,07	12,30	0,00	1,01	11,24	0,25	3,52	0,89	0,71
V1	1_NC	84,81	62,36	7,13	0,94	1,51	0,00	5,35	53,38	8,53	18,53	0,00	32,02	0,52	7,36	8,33	1,11
V2	1_NC	86,99	68,03	18,90	0,79	0,48	2,22	15,42	42,18	7,47	6,26	0,12	28,34	0,55	5,90	6,30	2,26
V4	2_PE	91,66	83,93	28,85	3,10	1,09	1,63	23,03	53,03	20,15	7,67	4,08	21,13	0,33	14,72	2,20	1,33
V3	2_PE	92,90	36,87	11,92	3,90	0,44	1,32	6,26	24,07	10,94	3,17	3,53	6,43	0,24	2,86	1,33	1,10
V1	2_PE	85,66	65,59	7,61	1,28	1,68	0,00	5,27	55,94	9,14	19,07	0,00	29,50	0,57	5,89	10,72	1,38
V2	2_PE	89,67	72,28	18,26	0,90	0,49	1,33	15,54	48,90	10,36	6,94	0,00	47,27	0,39	5,14	9,70	3,11
		-0,03	-0,05	-0,04	0,11	-0,45	-0,46	-0,03	-0,05	0,05	-0,23	-0,05	-0,08	0,33	-0,17	-0,02	-0,11
		-0,02	-0,32	-0,40	-0,76	-1,10	-0,40	-0,22	-0,27	0,10	0,00	-2,86	0,35	0,08	0,07	-0,63	-0,69
		0,02	0,02	-0,11	-0,09	0,15	0,00	-0,10	0,04	0,15	-0,23	0,00	0,02	0,19	0,53	-0,54	-0,62
**fold change**		-0,02	-0,08	0,06	-0,39	0,02	0,25	0,06	-0,17	-0,52	-0,50	0,99	-0,38	0,25	0,09	-0,23	0,03
to RPMI		0,03	0,02	-0,02	0,07	-0,17	-0,05	-0,02	0,04	0,12	0,03	-0,16	0,00	-0,18	0,06	-0,14	-0,11
		0,01	-0,08	-0,10	-0,09	0,00	-0,23	-0,10	-0,06	-0,01	0,01	-0,10	-0,14	0,04	-0,14	-0,09	-0,09
		0,03	0,07	-0,04	0,20	0,23	0,00	-0,11	0,08	0,21	-0,20	0,00	-0,07	0,26	0,41	-0,19	-0,30
		0,01	-0,01	0,03	-0,22	0,04	-0,26	0,07	-0,01	-0,09	-0,35	0,00	0,17	-0,05	-0,04	0,20	0,29

Sample	Condition	Gamma-Delta T cells	MAIT & NKT CD4- T cells	B cells	Naïve	Memory	Plasmablasts	NK Cells	Early NK	Late NK	Monocytes	Classical	Transitional	Non-classical	Dendritic cells	pDC	mDC
V4	0_RPMI	1,29	0,54	4,15	3,51	0,62	0,03	2,73	1,79	0,94	3,05	2,29	0,36	0,40	0,07	0,00	0,07
V3	0_RPMI	0,69	0,32	41,71	38,82	2,89	0,00	10,39	4,50	5,88	2,00	1,07	0,41	0,52	0,10	0,00	0,10
V1	0_RPMI	1,34	0,63	5,69	5,45	0,21	0,03	15,98	4,41	11,57	8,82	1,96	2,11	4,75	0,12	0,10	0,02
V2	0_RPMI	4,43	1,60	3,22	2,94	0,27	0,00	12,76	3,61	9,15	6,30	0,16	2,92	3,22	0,14	0,10	0,04
V4	1_NC	1,29	0,51	5,23	4,59	0,63	0,01	2,75	1,79	0,96	2,76	1,48	0,49	0,80	0,13	0,05	0,08
V3	1_NC	0,48	0,19	50,12	48,22	1,89	0,00	10,08	4,12	5,97	3,82	0,42	0,80	2,60	0,00	0,00	0,00
V1	1_NC	1,25	0,60	6,47	6,28	0,16	0,03	15,97	4,42	11,56	9,29	1,63	2,68	4,98	0,17	0,12	0,05
V2	1_NC	5,00	1,94	2,74	2,60	0,14	0,00	16,22	4,20	12,02	8,12	0,30	3,72	4,10	0,15	0,08	0,06
V4	2_PE	1,45	0,60	4,29	3,88	0,41	0,01	3,44	2,16	1,28	0,78	0,45	0,07	0,27	0,05	0,00	0,05
V3	2_PE	0,53	0,35	47,24	46,00	1,23	0,00	8,80	4,06	4,74	1,40	0,45	0,42	0,53	0,02	0,00	0,02
V1	2_PE	1,16	0,88	5,32	5,12	0,18	0,02	14,74	4,10	10,64	8,31	1,65	2,41	4,25	0,11	0,09	0,02
V2	2_PE	4,58	0,54	2,94	2,87	0,07	0,00	14,45	4,32	10,13	6,04	0,46	2,20	3,38	0,07	0,06	0,01
		0,00	-0,06	0,21	0,24	0,02	-2,00	0,01	0,00	0,02	-0,11	-0,55	0,27	0,50	0,46	0,98	0,13
		-0,44	-0,68	0,17	0,19	-0,53	0,00	-0,03	-0,09	0,02	0,48	-1,55	0,49	0,80	0,00	0,00	0,00
		-0,07	-0,05	0,12	0,13	-0,31	0,00	0,00	0,00	0,00	0,05	-0,20	0,21	0,05	0,29	0,17	0,60
**fold change**		0,11	0,18	-0,18	-0,13	-0,93	0,00	0,21	0,14	0,24	0,22	0,47	0,22	0,21	0,07	-0,25	0,33
to RPMI		0,11	0,10	0,03	0,10	-0,51	-2,00	0,21	0,17	0,27	-2,91	-4,09	-4,14	-0,48	-0,40	0,00	-0,40
		-0,30	0,09	0,12	0,16	-1,35	0,00	-0,18	-0,11	-0,24	-0,43	-1,38	0,02	0,02	-4,00	0,00	-4,00
		-0,16	0,28	-0,07	-0,06	-0,17	-0,50	-0,08	-0,08	-0,09	-0,06	-0,19	0,12	-0,12	-0,09	-0,11	0,00
		0,03	-1,96	-0,10	-0,02	-2,86	0,00	0,12	0,16	0,10	-0,04	0,65	-0,33	0,05	-1,00	-0,67	-3,00

Fold change = (Y – X)/X

X = Baseline (RPMI) values

Y = NC or PE values
